# Mechanisms of impact and contextual aspects of a dementia special care unit in long-term care: a process evaluation

**DOI:** 10.1186/s12877-021-02637-5

**Published:** 2021-12-07

**Authors:** Laura Adlbrecht, Sabine Bartholomeyczik, Hanna Mayer

**Affiliations:** 1 Department of Nursing Science, Faculty of Social Sciences, University of Vienna, Alser Strasse 23/12, 1080 Vienna, Austria; 2 Department of Health, Center for Dementia Care, Institute of Applied Nursing Sciences, FHS St. Gallen, University of Applied Sciences, Rosenbergstrasse 59, 9001 St. Gallen, Switzerland; 3 School of Nursing Science, Witten/Herdecke University, Stockumer Strasse 10, 58453 Witten, Germany; 4 Karl Landsteiner Private University Krems, Dr.-Karl-Dorrek-Strasse 30, 3500 Krems an der Donau, Austria

**Keywords:** Dementia, Special care unit, Nursing homes, Program evaluation, Theory-driven evaluation

## Abstract

**Background:**

In long-term care, persons with dementia are often cared for in specialised facilities, which are rather heterogeneous in regard to care concepts. Little information is available on how these facilities and care concepts bring about changes in the targeted outcomes. Such knowledge is needed to understand the effects of care concepts and to consciously shape further developments. This study aimed to explore the mechanisms of impact of a specific care concept from a dementia special care unit and the contextual aspects that influence its implementation or outcomes.

**Methods:**

Using a qualitative approach to process evaluation of complex interventions, we conducted participating observations and focus groups with nurses and single interviews with ward and nursing home managers. Data were collected from two identical dementia special care units to enhance the contrasts in the analysis of two non-specialised nursing homes. We analysed the data thematically. We conducted 16 observations, three group interviews and eleven individual interviews.

**Results:**

We identified seven themes in three domains related to mechanisms that lead to outcomes regarding residents’ and nurses’ behaviour and well-being. The themes include the development of nurses’ skills and knowledge, the promotion of a positive work climate, adjusted spatial structures, adjusted personnel deployment strategy “dedicated time for activities”, promotion of relaxation, of engagement in activities and of engagement in social interaction of residents. The implementation and outcomes of the care concept are influenced by contextual aspects relating to the (target) population and cultural, organisational and financial features.

**Conclusions:**

The study found expected and unexpected mechanisms of impact and contextual aspects. The care concept of the dementia special care unit results in higher levels of relaxation, activities, and social interaction of residents. Its implementation highly depends on the shared understanding of nursing and the skills of the nursing team. Changes in residents’ characteristics result in altered effects of the concept.

**Trial registration:**

DRKS00011513.

## Background

The majority of persons living in nursing homes have a diagnosis of dementia [1]. Their physical and psychosocial care needs are mostly complex, and in long-term care settings, they often experience a loss in their abilities, freedom, reduction in quality of life and avoidable health care service use, such as emergency department visits and hospitalisations that may be preventable by more appropriate nursing and physician care in the nursing homes [1–7]. Whereas the majority of persons with dementia living in nursing homes are cared for in non-specialised facilities, an increasing number live in dementia special care units [1]. Although there is no shared precise definition of dementia special care units (SCUs), an empirical study in Germany found that their main characteristics are a segregative living concept, special qualification in psychogeriatric care and additional funding [8]. Often, these aspects are complemented by a specific built environment and activities adopted to the residents’ needs [9]. Substantial research has investigated the impact of SCUs on residents’ functional status, neuropsychiatric symptoms, the use of restraints, psychotropic medications, quality of life, engagement in activities and social interaction [10–14]. Although these outcomes have been studied intensively, little is known about how they are achieved, especially as SCUs vary in their characteristics and interventions provided. Variations in characteristics and interventions include architectural features (e.g building specific for residents with dementia, use of exit control systems, number of rooms), financing (e.g. additional funding), professionals (e.g. special qualifications of nurses, continuous presence of registered nurse on night shift), residents (e.g. care degree, dementia diagnosis, number of residents who cannot be mobilized out of the bed, number of residents with psychological diagnosis), dementia policy and interventions (e.g. visitor regulations, recording of preferences, case conferences, evaluation of psychotropic drugs, use of dementia specific assessment instruments, presence of an expert on person-centred care) [15]. Evaluations of SCUs based on a specific theoretical understanding of their mechanisms of impact are lacking. Theories regarding the impact of the physical, social and organisational environment on persons with dementia stress the need for its congruity with a person’s abilities and needs [16–19]. However, the specialised care of people with dementia is complex and involves multiple players, structures and processes. Therefore, it is difficult to comprehend how the care concept influences outcomes; though, understanding this relationship is important to further develop specialised care for persons with dementia.

This study focuses on SCUs with a specific care concept and uses a theory-driven evaluation approach to understand the mechanisms triggered by the interventions and how they bring about changes in outcomes. The evaluation is based on a programme theory [20]. In a programme theory a theoretical understanding of the intervention is established by articulating assumptions about the intervention components, processes and desired, as well as undesired, changes [20, 21]. The initial programme theory includes a theory of action that describes the intervention components and a theory of change that describes the mechanisms and outcomes. The care concept is developed for persons with moderate dementia who demonstrate challenging behaviours. Its main features are as follows:

(a) Three small-scale, homelike wards for ten residents, including a kitchen and a living area at the centre, adjoining resident rooms and direct garden access;

(b) Educational interventions (including training in validation on Level 1 and 2^1^), coaching and supervision sessions for staff members;

(c) A person-centred and emotion-oriented approach in care using Naomi Feil’s Validation Therapy [23] that is based on the general principle of validation, the acceptance of the reality of another’s experience, unconditional appreciation and acknowledgement of the individual needs. Persons with cognitive impairments are classified in one of four stages on a continuum of dementia (Mal Orientation, Time Confusion, Repetitive Motion and Vegetation). Validation Therapy incorporates a range of specific communication techniques. The approach represents, on the one hand, an attitude in the care of persons with dementia and, on the other hand, it can be used as a structured therapeutic activity on an individual basis or in group sessions [24, 25]; and (d) Personalised non-pharmacological interventions. Challenging behaviour is addressed with relaxing, personalised, and emotion-oriented care to make the residents feel understood and to satisfy their needs. Activating, personalised interventions are used to promote social participation and engagement in purposeful activities. During the daytime, one nurse is specifically assigned to offer activities to residents (such as knitting, cooking, gardening, reading) in groups or at individual level.

According to the theory of change, the main outcomes of the SCU care concept are engagement in purposeful activities, social participation, feeling good at a place and have an impact on residents’ affective well-being. The initial programme theory guided the development of the methodology of the process and outcome evaluation [26, 27]. This article addresses the process evaluation of the care concept of the SCU.

## Methods

### Aim

This paper reports on the process evaluation of a care concept of the above-described SCU and aims to provide insights into how (mechanisms of impact) and under what circumstances (contextual aspects) the SCU triggers changes in outcomes, either intended or not, regarding residents’ and nurses’ behaviour and well-being. The results of the process evaluation will help to further understand the logic of the care concept, will check the plausibility of the assumptions described in the initial programme theory, will support the interpretation of the results of the outcome evaluation and will inform the revision of the programme theory. The described care concept was already implemented when the process evaluation started. Therefore, this paper reports only on the mechanisms of impact and contextual factors. The aim of this part of the project is translated into the following research questions:

- What mechanisms of impact (regarding residents’ and nurses’ behaviour and well-being) are activated by the care concept of the SCU?
- What contextual aspects influence the implementation of the concept and its outcomes?

### Design

The process evaluation of the care concept is based on two key functions of the Medical Research Council’s guidance for the evaluation of complex interventions: (a) mechanisms of impact and (b) contextual aspects [28, 29]. Mechanisms of impact intervene between the delivery of a programme and its outcomes and detail the responses of the target group to the interventions of the programme [28, 30, 31]. Contextual aspects of the care concept influence its delivery and/or functioning. Contextual aspects refer to “*circumstances in which an intervention is implemented that may interact with the intervention to produce variation in outcomes*” and include the organisational setting, cultural practices, characteristics of those delivering or receiving the intervention, political and financial factors [32]. In its guidance, the Medical Research Council suggests that the identification of mechanisms of impact is one of the most important functions of process evaluation and that these assumptions or as they call it “programme theories” are crucial to achieve an advanced understanding of the implementation and functioning of the intervention [29]. This understanding of process evaluation is consistent with the assumptions of Chen’s theory-driven evaluation about the function of mechanisms and context [20].

To examine the key assumptions of the initial programme theory and to explore how the programme works, we combined qualitative, deductive and inductive research approaches in our methodology. To incorporate contrasting perspectives that enhance the understanding of the sometimes-elusive mechanisms, we collected data with individual interviews, focus groups and observations in the SCUs that implemented the care concept and in non-specialised nursing homes (NSNHs). To address emerging questions during data collection and analysis, our data collection and analysis periods overlapped, and our data collection instruments were designed as being flexible enough to incorporate the emerging themes [28, 33]. All methods were performed in accordance with relevant guidelines and regulations, to structure our manuscript we used the Consolidated criteria for reporting qualitative research (COREQ) [34].

### Setting

The study was conducted in three nursing homes in rural regions of Austria. One of these nursing homes implemented the care concept under study in two SCUs, whose characteristics are described in the background section. The other two nursing homes are NSNHs where data were collected for contrasting purposes. Both nursing homes consist of three wards, with each being laid out for 40 to 45 residents with and without dementia. No specific care concept for persons with dementia is implemented. However, some nurses selectively and proactively offer isolated dementia care-specific interventions to individual residents. One to three nurses in each ward had been trained in Validation in a three-day basic course (see footnote 1). Furthermore, the nursing homes offer one to two planned group activities per day.

### Data collection methods

We conducted open and participating observations, focus groups and semistructured individual interviews. The observations were guided by the initial programme theory and focused on the mechanisms of impact of the interventions, the achieved outcomes regarding residents’ and nurses’ behaviour and well-being, and influencing contextual aspects. Residents with dementia and any intervention delivered to them were observed. Detailed descriptions of the interventions, the situations, the reactions of the residents and the context were included in the observation protocol.

In addition, we conducted focus groups with nursing staff members (herein referred to as nurses and representing all qualification levels of the nursing team: registered nurses, nursing assistants and home helpers), as well as semistructured individual interviews with ward managers and nursing home managers. To facilitate nurses’ reflections on mechanisms, we chose to interview them in groups where they could jointly elaborate on these mechanisms [35]. Conducting focus groups was not possible with the ward and nursing home managers because of their small number. Therefore, we conducted individual interviews with the managers. The development of the interview guides for the group and individual interviews were based on the initial programme theory and the results of the observations. This procedure enabled us to deal with topics arising in the observations or not answered therein (e.g., backgrounds for adaptions of interventions, observed deviations of the proposed mechanisms of impact). The interview guides addressed the following aspects: interventions focusing on nurses, spatial design and layout of the wards, activities and social interactions. For each aspect, the understanding, implementation, mechanisms and effects were discussed.

### Sample

For the observations, we recruited a purposive sample of residents who matched the target group of the care concept. We included residents who had a) a dementia diagnosis, b) regularly showed challenging behaviour and c) received or participated in interventions described in the initial programme theory. Other residents, staff members and visitors were observed when they interacted with the observed residents.

Focus groups were conducted with nurses of the participating nursing homes. Our experience with the development of the initial programme theory showed that nurses may struggle to describe the mechanisms of impact; therefore, we established a purposive sample of nurses who were known for their reflective strength. Further inclusion criteria were that the nurses had worked in the nursing home for at least 6 months and could express themselves well in the German language. Individual interviews were conducted with every ward manager and nursing home manager of the participating nursing homes.

### Procedures

Observation data were collected in two time intervals (7 am to 1 pm and 1 pm to 8 pm, each with two breaks of 20 min each). Both observation intervals were conducted once in every participating ward, which resulted in eight morning intervals and eight evening intervals. The observing researcher (LA) focused on 5–8 residents during one time interval. Potentially eligible residents were identified and approached by LA and the ward manager. Persons interacting with the focused residents were also observed. Observations took place in the common areas of the wards and in the bedrooms of the focused-on residents. LA tried to maintain a passive role by sitting quietly in the background, only interacting with others when directly approached and keeping interactions short. The observation protocols were handwritten and typed for digital data analysis.

For the individual interviews, the managers were asked directly for their consent to participate. The nurses participating in the focus groups were recruited by the ward managers. During each group interview, four aspects of the interview guide were discussed. In each of the individual interviews with the managers, the aspects corresponding to the expertise of the person were addressed. The focus groups and individual interviews were conducted by LA and TN in meeting rooms within the nursing homes and lasted between 45 and 90 min. The interviews were digitally recorded and then transcribed. Additionally, field notes were taken. Identifying information was replaced by anonymous wild cards (e.g. wild card instead of the real name - Mr. Miller = name of R8). Observations were conducted between March and June 2017, and focus groups and individual interviews were conducted between August and October 2018.

### Data analysis

**Fig. 1:**
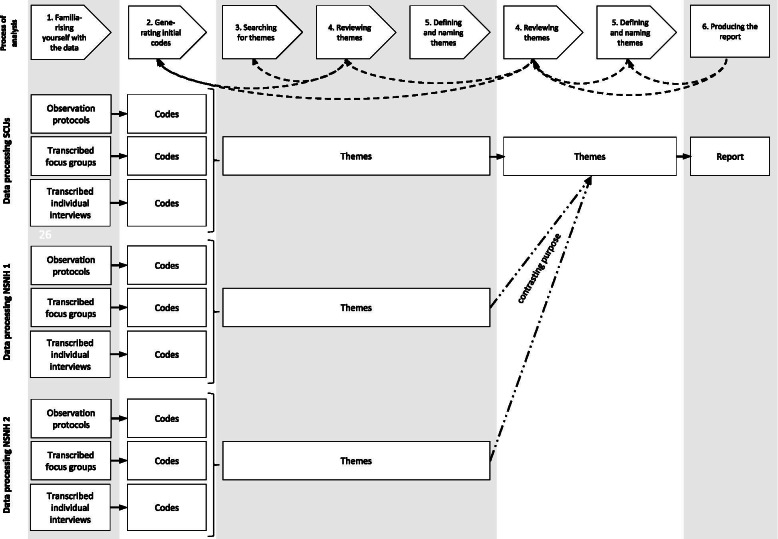
Process of data analysis and triangulation of data

The data analysis was organised with MAXQDA 2018 and followed the six steps of the thematic analysis by Braun and Clarke [36]. This method enables the identification of semantic and latent topics that are either developed inductively based on data or deductively based on the initial programme theory. First, we familiarised ourselves with the data, reread the transcripts and observation protocols and noted first ideas. In a second step, we systematically coded interesting aspects across the entire data set. Afterwards, (step 3), we looked for common aspects and connections between the codes and gathered them under potential themes. Afterwards, (step 4), we reviewed the consistency of the themes in relation to the coded text and the entire data set. This step had to be done repeatedly as we identified interesting new text passages or codes and themes that needed to be rearranged. Subsequently, (step 5), the themes were refined and named, and relationships between them were described. In the sixth step, we produced the report and a tabular presentation of the themes. Since we included several types of data from different settings in the analysis, we triangulated them in a multi-stage process (see Fig. 1). For each text (observation protocols and interviews), steps 1 and 2 were conducted separately. Steps 3 to 5 were conducted for all the data from one nursing home considering the varying statements in different data sets. Subsequently, we brought the themes from the analyses of the individual nursing homes together by carrying out steps 4 and 5 again for the entire data set. Therefore, we focused on the care concept of the SCU and used the results from the other two nursing homes to contrast them and to gain better insight into the mode of action and mechanisms of impact of the care concept. The coding was performed by LA, and codes and themes were critically discussed in multiple analytic sessions with the co-authors and two further nursing scientists until we reached consensus.

### Ethics

The ethical board of the German Society of Nursing Research approved the study (No. 16–024).

Consent for observations was obtained from all observed residents and all persons possibly interacting with them (e.g. nurses and other residents) before the observation started. Interactions with persons who did not agree to participate in the observations because they did not want to or because there was no opportunity to obtain informed consent beforehand (e.g., visitors who suddenly appeared or nurses from other wards) were not protocolled. To ensure that all participating persons with dementia were able to decide on the highest possible level of information and understanding, they received information about the study adapted to their abilities. Informed consent was obtained from all persons with dementia with the capacity to consent tested with the German version of the University of California, San Diego Brief Assessment of Capacity to Consent [37]. If persons with dementia were not capable to consent, their affirmative agreement was expressed either verbally or non-verbally (assent) [38] and, if designated, their legal representatives’ informed consent was obtained. Assent and informed consent were established at the time of enrolment in the study and revisited at each observation session (ongoing consent). Informed consent was obtained from all participants of the focus groups (nurses) and individual interviews (managers).

### Trustworthiness

To enhance trustworthiness, multiple strategies were applied. We triangulated data from different data collection methods and from different sites to test and contrast data and their interpretation. To ensure that participants’ statements were accurately captured and to verify our interpretation, we conducted a member check by discussing the results with nurses and managers of the SCUs. The translation of the quotes embedded in our article was double-checked by LA and a nursing scientist native in German and English. We know the care concept well, and to reflect our prior knowledge and assumptions critically, we performed parts of step 2 of the thematic analysis [36] in analytical sessions with nursing scientists who were not involved in the study. All researchers have experience with qualitative studies and in research with persons with dementia.

## Results

The results of the process evaluation of the care concept are based on 16 participating observations of 49 residents and 76 persons interacting with them on the wards of the SCUs and the NSNHs, three focus-group interviews with 17 nurses, and eleven individual interviews with ward and nursing home managers (Table 1).

**Table 1:** Sample description

Data source	Total	SCU	NSNH1	NSNH2
**Observations**				
**Number of observation units, n**	**16**	4	6	6
Number of observation units in the morning, n	8	2	3	3
Number of observation units in the afternoon, n	8	2	3	3
**Number of residents focused on in the observations, n**	**49**	16	15	18
Gender, n women	38	12	13	13
Age, median (range)	82 (60–98)	82 (62–98)	83 (60–97)	81 (65–95)
MMSE score, median (range)	14 (0–21)	15 (8–18)	13 (0–21)	14 (3–20)
**Number of persons observed interacting with the residents focused on in the observations, n**		17	27	32
Residents, n	**76**	9	9	11
Nurses, n	29	7	15	20
Visitors (e.g., family members), n	42	1	3	1
	5			
**Interviews**				
Number of individual interviews, n	11	3	4	4
Number of focus groups, n	3	1	1	1
**Number of persons participating in individual interviews, n**	**11**	3	4	4
	3	1	1	1
Number of nursing home managers, n	8	2	3	3
Number of ward managers, n **Number of persons participating in focus groups, n**	**17** 5	6 2	5 2	6 1
Number of registered nurses, n	11	4	3	4
Number of nursing assistants, n	1	0	0	1
Number of home helpers, n	14	0	2	1
Gender, n women	45 (25–57)	49 (38–54)	45 (28–57)	39 (25–52)
Age, median (range)	9 (3–31)	11 (8–14)	10 (4–31)	8 (3–22)
Professional experience in years, median (range)				

**Fig. 2:**
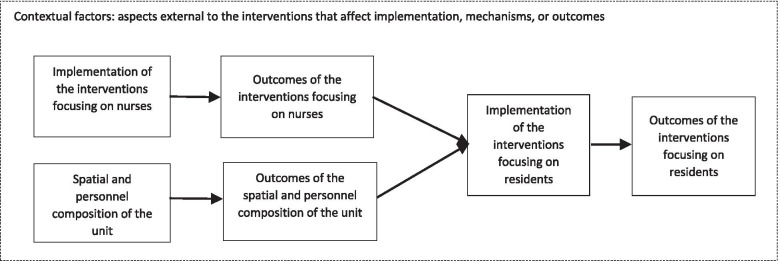
Relation between interventions and contextual aspects influencing implementation and outcomes of the care concept

The results regarding the mechanisms of impact were organised into three domains and seven themes. In addition, six contextual aspects influencing implementation and mechanisms of impact of the care concept were identified (see Table 2). The interventions for residents were found to depend on the successful implementation of the interventions for nurses and the spatial and personnel composition of the unit (Fig. 2). This dependency is characterised by the need to develop nurses’ skills and knowledge to empower them to carry out interventions for residents. In addition, a positive work climate, spatial structures and an appropriate personnel deployment strategy enable nurses to actually provide interventions to residents adequately. Furthermore, contextual aspects influence the provisioning of the interventions.

**Table 2:** Mechanisms of impact of the care concept of the SCU

Domain	*Theme*	Intervention	Mechanism	Outcome	Impact
*Interventions focusing on nurses*	*Development of nurses’ skills and knowledge*	Training team members in validation	Shapes the understanding of nursing as well as of dementia and its impact	Adjusted care practices towards an adequate response to the needs of residents	• Altered prioritisation of care tasks • Altered time management
		Discussion in the team of situations experienced as problematic or challenging by individual nurses	Enables team members to reflect their experiences together and express their opinions	Creative and innovative solutions for situations experienced as challenging	Continuous acquisition of competences of the whole team and continuous improvement in self-efficacy of the whole team (positive reinforcing feedback loops)
		Providing all nurses access to the same training	Shapes a shared understanding of and competence in care for persons with dementia	• Team competence and self-efficacy: the ability and experience of every nurse to react to changing situations • Shared and consistent approach to care	Flexibility in care
	*Promotion of a positive work climate*	Joint discussion of situations experienced as problematic or challenging by individual nurses in the team to jointly find solutions	• Promotes a mutual understanding and a feeling of being a valued team member • Makes the nurses feel not to be left alone with a problem • Enables the provision of good care, also in situations experienced as challenging	• Positive work climate • Provision of good dementia care • Culture of solidarity: nurses are looking out for and support each other	• Promotion of staff retention • Sustainable implementation of the care concept
		Informal and formal team gatherings during and after work	Promotes mutual appreciation and team cohesion		
*Spatial and personnel composition of the unit*	*Adjusted* *spatial structures*	Small-scale, household-like units	• Are perceived as spaces with a constant but low level of acoustic and visual stimuli • Facilitate the fulfilment of the needs of engagement in activities and social life as well as withdrawal	• Residents spend most of their time in common areas • Residents seldom retreat to their bedrooms	• Social engagement • Engagement in activities
	*Adjusted personnel deployment strategy – “dedicated time for activities”*	Extra nursing shift dedicated to promoting activities	• Provides nurses with time to promote activities and social interaction • Conveys nurses the feeling, that the promotion of activities is part of their job	• Establishes an understanding of nursing within the team that includes physical, psychosocial and occupational tasks directed at the individual persons preferences, desires and needs • Promotion of residents’ engagement in activities and social interaction by nurses of the extra and the “normal” shifts	• Social engagement • Engagement in activities
*Interventions focusing on residents*	*Promotion of relaxation*^a^	Personalised psychosocial interventions directed at the individual persons preferences, desires and needs at an early stage of agitation or to prevent agitation or other challenging behaviour	• Leads to relaxation of the specific resident	• Relaxed, purposeful actions of the specific resident • Social engagement • Engagement in activities	• Relaxing, calm, peaceful environment • Time spent in communal areas • Work processes of nurses
	*Promotion of engagement in activities*	Activities offered by nurses throughout the day personalised in content, type, timing, duration and participation mode (single or group, self-initiated with support from nurses, initiated by nurses with active participation, initiated by nurses with active, supported participation or initiated by nurses with passive participation)	• Increases the motivation for participation • Promotes residents` focus on the activity • Enables residents to use their resources purposefully • Enables residents to interact meaningfully with the environment and the people in it	• Time spent on activities • Positive experiences	• Social engagement • Relaxation
	*Promotion of engagement in social interaction*	• Constant, personalised impulses for the social interaction of nurses and the living in a household-like unit • Active, appreciative communication with family members	• Promotes spending time together • Promotes shared experiences • Promotes social interaction between residents • Promotes social interaction between residents and nurses • Makes family members and loved ones feel valued and welcome	• Becoming part of a social community • Involvement in a fragile, social (sub-)group • Emotional connections to others • Relationship with family members and loved ones	• Belonging • Relaxation • Affection

^a^ Definition of relaxation: In the interviews, nurses and managers agreed that relaxation was understood as an opposite state of agitation and in distinction from apathy. They recognise relaxation in a low muscle tone, relaxed neck and face muscles, a focused look, the ability to stay calmly at one place, the absence of repetitive movements and intense (negative) feelings and unmet needs. A significant characteristic of relaxation is seen in the contact ability that is described as residents’ ability to react in an adequate way to interactions of others enabling them to communicate with them

### Interventions focusing on nurses

#### Development of nurses’ skills and knowledge

The development of nurses’ skills and knowledge is based on the training of all team members and on collective learning through problem discussion and solving within the team and thereby increases team competence and results in altered and shared care practices (Table 2).

Validation training primarily shapes the fundamental attitude towards care that is expressed by the validation of emotions and enhances the amount of empathy shown during care for persons with dementia. Nurses were seldom observed using validation techniques with agitated residents. The focus group participants considered the validating attitude towards care in combination with systematic biographical work as enablers to recognise residents as persons with individual needs, resources, and preferences. Subsequently, nurses described that they are able to orientate their care on the persons’ needs, resources and preferences and thereby are able to respond adequately to unmet needs. The observations showed that nurses in the SCUs, compared to those in the NSNHs, use their time resources differently by spending more time on the promotion of activities and social interactions and needing less time to respond to distress and agitation.

Apart from formal training, nurses described that they acquire skills and knowledge by discussing challenging situations with their team. This process was regularly observed with residents showing persistent vocalisations or efforts to run away. Nurses from the SCUs and NSNHs outline their experienced difficulties in informal team gatherings, reflect on their experiences together, discuss opinions and find solutions. Therefore, the team continuously expands and revises its knowledge and skills in dementia care. In an individual interview, a manager described this as follows:

*“So that (.) if there are any problems that are very stressful, you also include the team, because the solution usually comes from the team anyway, I have to say. Because if it is a larger group and you discuss, for example, any problem that arises, (.) either we or an individual colleague find a solution.”* (EI11_58).

As all nurses experience the same development of skills and knowledge, they described that they are more likely to act in synchronisation and thereby offer a consistent approach to care.

*“You will make a big leap if you have completed validation training. In addition, above all, as EVERYBODY is trained, we learned to think in the same way.”* (GI1_28).

As all nurses are trained, everyone has the capacity to react adequately in changing situations, e.g., when a resident acts like she or he never has before. Residents’ behaviour was observed to change suddenly and unpredictably; therefore, this team skill is especially useful for preventing stress and agitation. However, the observations in the NSNHs with only a few trained team members revealed that stressful situations may be prolonged if the trained nurses are not on-site.

#### Promotion of a positive work climate

A positive work climate is enhanced by collective problem solving, informal and formal team gatherings and mutual appreciation and thereby contributes to the sustainable implementation of the care concept.

The focus group participants reported discussions of difficult situations with the team to jointly find solutions. The discussions made them feel understood, appreciated and not left alone to deal with a problem. They described formal and informal team gatherings as being important for team building and team cohesion. Both problem discussions and team gatherings contribute to a positive work climate and a culture of solidarity, where team members support each other in particularly challenging situations.

*“Well, taking care of each other and reminding each other to take time-outs, for example, helps a lot. If there is someone who says, “Let us go smoke one,“ this only takes five minutes, and then it’s alright again. Many of us look out for each other.”* (EI7_100).

Furthermore, mutual support and jointly developed solutions for particularly challenging situations enable nurses to be more likely to provide good dementia care. The positive climate on the team, the feeling of appreciation and the ability to provide good care reduce nurses’ distress. According to the managers of the SCU, this can be seen in high staff retention rates. Staff retention leads to increased efficiency of training measures and higher team competence, which is essential for the sustainable implementation of the care concept.

### Spatial and personnel composition of the unit

The design of the unit and the personnel deployment strategy create spaces, times and an understanding of nursing that promotes activities and social engagement (but also enables privacy and withdrawal) (Table 2).

#### Adjusted spatial structures

As pointed out in the interviews with the ward managers, the household-like design of the unit offers spaces that support the fulfilment of residents’ needs (e.g., a spatial layout that enables tacit orientation, a patio and a garden with safe walking opportunities, a dining and living area for eating, recreational activities and social life, various sitting options in the indoor patio for tranquillity and resting, a bedroom for privacy). They see a major advantage in the fact that all parts of the unit are in direct sight of the residents and easy to access, thereby enabling residents to autonomously navigate through the unit according to their preferences, desires and needs.

*“The background was actually that we can give the residents an intimate space where they can easily find their bearings. That means having FEW people to deal with. In the middle, a living/dining room surrounded by bedrooms. That is, when the resident comes out of his or her bedroom, he is in HIS living room. On a conventional ward, you come into the aisle and must orientate yourself, ‘Do I go left, do I go right?’ In addition, we just wanted to prevent this additional disorientation.” (EI3_4).*

The observations yielded a constant but low level of acoustic and visual stimuli in the small-scale units. In the larger units of the NSNH, however, the level of stimuli (e.g., noise level) was noticeably higher, and residents left situations with extremely high levels of stimuli (e.g., mealtimes) quickly. Residents were seen spending most of their time in areas with a comfortable level of stimuli and with opportunities for the fulfilment of their needs (e.g., social interaction in common areas).

#### Adjusted personnel deployment strategy “dedicated time for activities”

In addition to the normal nursing shifts, the development team of the care concept established an extra shift, within which nurses’ sole assignment is the provision of occupation either in groups or for individual residents. The individual and focus-group interviews with the members of the SCUs show, especially in contrast to the interviews with the members of the NSNHs, that they integrate not only physical and psychosocial nursing care but also the provision of meaningful activities into their understanding of nursing. The change in the understanding is reflected by nurses who enhance engagement in activities in the extra shift and in the normal shifts.

*“Are they going to do something by themselves? No. WE have to give them something and then work with and watch them… (name of resident), for example, she does not stimulate herself, so I sit down, ask her something and she blooms while telling me her stories.” (GI1_43).*

An unintended outcome of the personnel deployment strategy is that nurses recognise residents’ great need for engagement in activities and social interaction. The interviewed nurses of the SCUs consider the promotion of engagement in activities and social interaction as their task, and even though they try to use their time optimally, they cannot offer it on an ideal level for all residents. Consequently, they describe having a certain level of distress as they must decide who is going to experience activity promotion and who is not.

### Interventions focusing on residents

The themes a) promotion of relaxation, b) promotion of engagement in activities and c) promotion of social interaction present interlinked bundles of interventions (Table 2). On the one hand, they are interlinked because sometimes the same intervention can provide relaxation, engagement in activity and/or social interaction. An example is a group singing activity accompanied by social interaction that promotes residents’ relaxation if it meets their needs. On the other hand, the three complexes are interlinked via their achieved outcomes. Because residents are unique individuals, they respond differently to interventions, allowing the same intervention to achieve different outcomes and different interventions to achieve the same outcomes. Therefore, the three intervention complexes promote the same outcomes, especially relaxation, social engagement and belonging and seem to contribute to the overall well-being. In addition, all three intervention complexes are provided in a personalised manner, meaning they are tailored to residents’ needs, resources and preferences.

#### Promotion of relaxation

For the implementation of the promotion of relaxation (definition in Table 2), nurses primarily use an emotion-oriented approach with psychosocial interventions referring to the individual persons’ preferences, needs and desires. These interventions are provided as soon as nurses detect a potential for stress, agitation or apathy, but they are also applied for de-escalation purposes. Such interventions allow residents to relax, become contactable, and participate in purposeful activities and social interactions.

Subsequently, the interventions support a calm and relaxed atmosphere on the wards, which was only selectively interrupted by arising restlessness during the observations. Nurses reported that residents usually reflect on the conditions or behaviours, be they calm or agitated, that lead to a reinforcement of the relaxed atmosphere on the ward. A registered nurse described such reinforcement, this time negatively, in the following quote:

“*When you come to the ward and you are out of balance, you can be sure that the group is EQUALLY out of balance all day long. They have sensors for that, and if you’re having a bad day, you’re already screwed. In addition, they notice exactly when you’re sad, they look at you and ask, ‘What’s wrong?’ even though you do not do anything differently and think that you’re acting quite normal. It works the other way around too; if you come in a good mood, the residents reflect this too.*” (GI3_161).

The calm atmosphere is reflected by the low acoustic level, calm movement patterns and purposeful actions of the residents. Supporting the assumption that residents like the atmosphere in common areas, residents were observed spending most of their time there, and withdrawal to one’s bedroom was seldom observed in the SCUs. In the NSNHs with a higher noise level, residents with and without dementia tend to seek the quietness of their rooms and use them for relaxation and recreational activities during the observational periods. However, some residents seemed comfortable in high-noise situations that others tended to find stressful – an aspect that reflects the residents’ uniqueness.

The observations in the SCUs that contrasted with the observations in the NSNHs showed the early promotion of relaxation results in a lower amount of time needed for calming and reassuring the residents and preventing the development of a negative social dynamic that would in turn affect other residents. Nurses are therefore likely to invest their gained time resources in the promotion of activities and social engagement, which meet residents’ needs and further enhance relaxation. In these situations, a positive social dynamic enhancing relaxation and fulfilment of needs could be observed.

#### Promotion of engagement in activities

Interventions to promote engagement in activities are provided in organised weekly planned group activities by the elder-care team^2^ and as individual activities by nurses, thereby allowing residents to experience activities throughout the day. During the observations, nurses of the SCUs provided activities to individuals or small groups that were individualised in regard to content, type and duration to meet the physical, cognitive and social abilities, as well as preferences, of the residents. In the interviews, nurses described that this type of activity promotion allows residents to experience activities that they enjoy. This not only creates a positive experience for the residents but also promotes relaxation and focus on the activity, the use of their skills and interaction with their environment.

“*They are calmer, they talk much more about it. They also make statements that you would not expect at all, that you do not believe that they still know about. They truly behave differently. They are more communicative, in any case. (.) You have the feeling that they are calmer inside. It is not about their appearance, the fidgeting or something like that, but you have the feeling that they truly give you their full attention and they are calmer for themselves.”* (EI4_67).

Group activities are usually not or less personalised. The observations showed that the reaction of the residents depended on the extent to which the activity offered by the elder-care team corresponded to their needs, abilities, and preferences.

Nurses reported that the effects of activating interventions are usually limited to the time of the activity. An impact on residents’ mood, in the sense of verbal expressions, facial expressions or behaviour, was only reported in a few cases. It is questionable, however, whether there truly was no change in mood or whether it was difficult to determine because of the impact of dementia on emotional expression. Often, the mechanism of impact is triggered by an interaction initiated by nurses that is modulated on top of an activity, thereby increasing its scope and impact, as described in the following observation protocol:

*N12 turns on the TV and helps residents make themselves comfortable in front of it: “What do you say we watch series XY now? Yesterday there was (...); let us see how things go on today. What do you think, (name of R8)? Would you like to watch too, (name of R2)? Well then, come and join us. Shall we bet that they kiss today? All those in favour?” “Sure, they will!” (says R8) Two residents lift their arms. “Who’s against it?”* (B14_p. 6).

#### Promotion of engagement in social interaction

Nurses promote engagement in social interaction by starting and guiding conversations or by setting off impulses that are likely to trigger social interactions. Social interaction helps residents to become part of a social community by facilitating a sense of belonging. Becoming part of a social community also happens passively when residents have common experiences that create an emotional connection. This is especially true for residents with advanced dementia, whose opportunities for verbal interaction may be severely limited. The observations made in both settings yielded that the social community is reflected by residents knowing each other and demonstrating behaviours that characterise the social conventions of social groups (e.g., greeting all present when entering the living room, saying goodbye before going to bed, sharing the newspaper and discussing the content, commenting on the weather).

The focus group participants reported that sometimes within the ward, smaller subgroups of two to four people are formed that are primarily based on mutual positive perceptions. The emotional ties of these residents are closer, which is reflected by residents being keen to spend time together (e.g., walking around together, sitting next to each other for certain activities) and by increased interaction between these residents:

“*Therefore, for example, we now have a group that has just formed itself. It is an insanely intense group. They’re having a great time. Even the residents, who nobody had accepted before. There were always two ladies who nobody accepted. Now suddenly, the group works with six residents at the table. In addition, each of the ladies demands her own topic of conversation*.” (GI2_186).

In such subgroups, the residents experience mutual affection, which sometimes manifests itself physically through a hug or stroking of the hand. However, the subgroups are described by nurses as fragile entities that.

*“(…) can dissolve unpredictably. In our ward, it went well for a while when two ladies helped to set the table. Then, one of the two ladies got worse and the second one said, ‘Well, I am not going to do that alone.’ And since then, she just sits there and looks. That’s because she did not want to do it alone*.” (GI2_76).

The promotion of social interaction includes the facilitation of (close) relationships with family members and loved ones. Nurses promote these exchanges with family members; they openly communicate information about the residents and seek the expertise of family members on, for example, the biography of the residents. In this way, family members feel valued, welcome, and integrated and tend to visit residents more often or for longer periods.

“*My experience is that if we take the relatives on board right from the start and we are very open in what we say and do [...], I actually have the relatives very much on board, they help out, they deal with the biographical work of their own dad, mum or whatever. In addition, that leads them to truly deal with them. You see that. They simply visit longer, inform themselves about their family member, what happened, how he is?*” (IG_EI1_33).

### Contextual aspects

Seven aspects within the context features population, cultural, organisational and financial are found to influence the implementation and outcomes of the care concept. The aspects and corresponding quotes are displayed in Table 3.

**Table 3:** Contextual aspects influencing implementation and outcomes of the care concept

Context aspects [32]	Topic and its short description	Influence on implementation and/or outcomes of the care concept	Quotes or/and extracts from observation protocols
*1. (Target) Population*	*Persons with severe physical impairments and/or severe dementia live in nursing homes* Living at home even with (more) severe physical and cognitive impairments is possible due to increased care competence of formal and informal caregivers Leads to later admissions to nursing homes and changes in resident characteristics	Increased need for support in physical care needs leaves less time to implement the promotion of activities and social interaction	“That is what I believe is the problem, that the residents simply come to us with high physical care needs, that we can no longer promote the resources as it is stated in the care concept. In addition, of course we have enough staff for the way this concept should be, but as it looks now, we do not have it anymore and therefore team members are simply and constantly, massively overloaded and frustrated.” (EI3_89)
*2. (Target)Population*	*Constant behaviour changes possible* Persons with dementia unpredictably change their behaviour and reactions to interventions, persons or situations Nurses must be able to manage changing situations at any given moment and therefore all nurses need to have the appropriate skills and knowledge	High need for a stable team competence and early, intensive training of new team members, if this is not achieved implementation of interventions focusing on residents is at risk	“With dementia, I will say one thing is that there is no common thread. That is the real tension.” (EI2_63) “When I come in the morning, I never know - yesterday it was great, and today nothing works. You always have to adjust and make the best of it.” (IG_GI2_ 75)
*3. (Target)Population*	*Family members as advocates* As persons with dementia experience difficulty fully recalling their biography and comprehensively assessing situations, family members take on the role of advocates	Need for intensified cooperation with family members, as they have essential information for residents’ care and on the other hand need information to be able to act as advocates, affects implementation of the care concept	“My experience is that if we take the relatives on board right from the start and we are very open in what we say and do [...], I actually have the relatives very much on board, they help out, they deal with the biographical work of their own dad, mum or whatever. In addition, that leads them to truly deal with them. You see that. They simply visit longer, inform themselves about their family member, what happened, how is he or she?” (IG_EI1_33)
*4. Cultural – attitude of the target population*	*Intolerance of residents towards behaviour that does not meet social conventions:* Residents without, with mild or moderate dementia address behaviour that does not meet social conventions in a punitive manner	Intolerance of socially unsuitable behaviour by persons with dementia influences the outcomes of the care concept of belonging to a social community	Note at 10:40 a.m.: R14 goes to the next table and drinks from the glass of another resident, whereupon R8 shouts: “Are you stupid? This is not your glass.” […] Note at 10:55 a.m.: R8 sits down on an armchair that is not his, another resident starts to nudge him with a stick until R8 gets up and leaves grumbling and grumpy. (B3_p. 5)
*5. Cultural - belief of the managers and practitioners*	*Empathy as a must-have in the care for persons with dementia:* For the delivery of interventions focusing on residents, empathy is believed to be essential Empathy is regarded as a characteristic that is difficult to train	Conscious recruitment of new team members difficult because of structurally small number of nursing staff on job search and may jeopardise the implementation	“When we hire someone new, the most important thing for me is that he or she deals empathically with the residents. We look at this by letting them spend two days on the SCU. The staff then gives me feedback on whether he or she fits into the team. I pay less attention to formalities - everything else can be learned in trainings.” (EI6_34)
*6. Organisational*	*Transformational, consistently acting leaders shaping the organisational culture and understanding of nursing* Charismatic leaders convinced of their approach to care were seen to inspire nurses and to empower them to act in the same way	Common, person-centred attitude in interaction with each other influences the work climate and care practices Changes in leaders and personnel may lead to changes in the informal organisational culture and thereby may influence the implementation of the care concept	Expression of common person-centred attitude and conformity: “We try to determine, what resident like. We look at the biography, where were the interests thus far, talk to the relatives, what they did at home until the end, but you are not allowed to forget that people change. If she was knitting for 40 years, then she often does not want to know anything about knitting anymore. ‘I have knitted long enough, yes, I do not like it anymore - I want to do something new.’ Yes, so people with dementia not only want to do the same old things.” (EI1_18) “Exactly, and our experience is that relatives say in regard to the biography “Yes, they have always loved having children around” and so on, then we ask them, and they can’t stand it. People change, especially with dementia.” (EI3_6) Example of conformity: N4: “If you’re nervous, it is over.” N6: “If you’re having a bad day.” N4: “You’re already lost.” N5: “Because they know that [yes], they pick that up right away. Then, your whole day is gone” (laughs). N4: “They feel that. [Yeah, right]. Sensors they have. Sensors, they already have good ones. Whenever something is...” N6: “So the feeling.” [IG_GI2_161–166]
*7. Financial*	*Limited time resources in nursing care:* Time resources are limited and are particularly scarce during holiday periods and periods of high sickness absence	Limited time resources influence the implementation of the interventions focusing on residents Limited time resources sometimes collide with the developed understanding of nursing resulting in a prioritisation of tasks in favour for physical needs and frustration of nurses	“The time you need, you should have; it is very important that you can take your time. Good care needs time.” (GI1_7) “Five things are going on at the same time. The bell rings, someone shouts, the ward round comes... In addition, then you cannot arrange anything with the residents with dementia. You cannot say to them, ‘Stay here for five minutes, I will be right back.’ They do not know what five minutes is. You have to act immediately.” [VG_EI6_87]

With regard to the target population, the managers of the SCUs and NSNHs reported that the population is changing (contextual aspect 1), with more persons with severe dementia, high levels of physical care needs, (severely) limited mobility and psychological symptoms or diagnoses living in nursing homes. Furthermore, they pointed to the increasing number of persons showing disinhibitory behaviours. This population differs from the target population of the care concept, which was originally developed for persons with moderate dementia who demonstrate challenging behaviours, can move around on the ward independently and perform light household tasks. The managers see possible reasons for these changes in the increased competence in home care, which enables persons to stay longer in their own home. For the SCUs, this means fewer residents who correspond to the defined target group, more time needed for physical care and less time available for the promotion of engagement in activities and social interaction. In addition, the increased physical and cognitive impairments hinder the promotion of engagement in activities and social interactions in groups requiring individual, time-intensive offers. Another aspect of the target population reported in both settings is that people with dementia often suddenly change their behaviour (contextual aspect 2), which makes the strict adherence to care plans difficult. Nurses must be able to manage changing situations at any given moment. If nurses lack this competence, the unpredictable actions of persons with dementia result in distress for both nurses and residents. An additional aspect of the target population is that due to their impaired memory and decision-making capacity, family members act as their advocates (contextual aspect 3), which results in intensified cooperation between family members and nurses.

A cultural aspect of the context observed in both settings is the intolerance of residents towards the behaviour of other residents that is perceived as not being in line with certain social conventions (contextual aspect 4). If a behaviour disturbs residents, they comment on it, sanction it and try to stop it. The observations yielded statements that were often coarse, insulting for the persons referenced and sometimes aggressive. Intolerance towards other residents’ behaviours was observed in persons with no, mild, and moderate but not severe dementia. Another cultural aspect is the belief of nurses and managers of the SCUs that care for persons with dementia requires a basic level of empathy (contextual aspect 5), which is difficult to train and essential for the implementation of care based on a person’s needs, resources and preferences.

An organisational aspect of context is the transformational leadership style shown by the SCU managers (contextual aspect 6). They are convinced of their approach to dementia care and live their underlying person-centred values in the interactions with residents and nurses, be it in everyday conversations, in organising roasters or in providing support in stressful times. In the observations and interviews, they showed a strong, charismatic appearance and were perceived by the nurses as figureheads. These leaders shape nurses’ understanding of respectful interactions with each other and of the care of persons with dementia. In the interviews and in the observations, nurses and managers spoke and worked together consistently, and they were convinced of and enthusiastic about their approach to dementia care. This approach influences the work climate and contributes to a stable team that consistently cares for residents with the same attitude in direct care.

A financial aspect of the context under which care in nursing homes takes place is the limited time resources (contextual aspect 7). Nurses describe having an inner conflict when time resources collide with the developed understanding of nursing that is inherent to the care concept. In such situations, nurses are frustrated and prioritise tasks related to physical needs.

## Discussion

The results of the process evaluation provide insights into how and under what circumstances the care concept of the SCUs triggers changes in outcomes. The findings point to aspects of the initial programme theory that require modification. Table 4 provides an overview of the most substantial indications for the revision. Although the results are not transferable to other settings, as they are linked to the specific care concept and context, the mechanisms of parts of the care concept may also apply in other concepts and in other settings.

**Table 4:** Major indications for the revision of the initial programme theory as implied by the results

Domain	Theme	Major indications for confirmation, extension and modification of the initial programme theory as implied by the results of the process evaluation
*Interventions focusing on nurses*	*Development of nurses’ skills and knowledge*	Confirmation: interventions for nurses increase their competence and communication skills Modification: interventions for nurses do not reduce the strain related to dementia care but do increase self-efficacy Extension: an increased competence of the entire team is needed so that nurses can respond flexibly and adequately to changing resident behaviours
	*Promotion of a positive work climate*	Extension: a positive work climate is regarded as being essential to maintaining a stable team, which is needed for sustainable implementation
*Spatial and personnel composition of the unit*	*Adjusted spatial structures*	Confirmation: spatial layout reduces stress among residents Extension: spatial layout promotes engagement in activities and social interaction
	*Adjusted personnel deployment strategy “dedicated time for activities”*	Extension: the important role of the personnel deployment strategy in shaping the understanding of nursing and facilitating its implementation only became clear in the process evaluation; all findings regarding this theme complement the initial programme theory
*Interventions focusing on residents*	*Promotion of relaxation, engagement in activities and social interaction*	Confirmation: interventions focusing on residents promote relaxation, engagement in activities and social interaction Modification: interventions focusing on residents, their mechanisms and outcomes are strongly intertwined; for example, the same intervention can promote relaxation and engagement in activity but can be experienced differently interindividually

Quality of care is influenced by nurses’ behaviours that are shaped by the organisational culture and personnel strategy, emphasising a need for sufficient, knowledgeable and skilled personnel [39, 40]. Interventions to promote nurses’ expertise and to develop an organisational culture are found to be effective only with a relatively stable team. Staff retention is higher and knowledge drains are lower in supportive workplaces, including adequate staffing levels and working conditions, as well as management support and a person-centred care environment [41–45]. These are aspects to consider, as high staff retention is a key ingredient for the sustainable implementation of the care concept.

In research initiatives, the role of the built environment is often discussed in connection to wayfinding [46] influencing residents’ safety and ability to perform activities of daily living, such as toileting or dressing [47]. The findings of the process evaluation indicate that a dementia-sensitive spatial design additionally has an impact on residents’ social life and their engagement in purposeful and leisure activities, as also shown by Lee et al. [48] and Morgan-Brown et al. [49].

A core element of the care concept is the promotion of activities and social interaction, which address a major need of persons with dementia who want to be involved in activities [50]. The care concept follows the imperative of person-centeredness in dementia care to the extent that the needs, preferences and resources of the residents guide the interventions; however, since it is not systematically implemented at the organisational level, the concept depends on the initiatives of individuals, thereby making it vulnerable to staff or organisational change, which means that it may only contribute to a limited extent to maintaining personhood and continuous thriving [51–53]. Even though the care concept aims to increase engagement in activities and social interaction, we do not know how much time spent with activities or social interaction each person prefers. Sometimes times of “doing nothing” can be experienced as being relaxing, restorative and especially valuable to residents [54]. Our data are supported by a review of interventions for loneliness showing that the promotion of relaxation, social interaction and activity are intertwined and that each aspect contributes to the others [55].. Furthermore, persons with dementia only marginally seize opportunities to engage socially with others and often rely on nurses to facilitate interactions [56]. Therefore, an understanding of nursing considering the promotion of social interaction as part of nursing contributes to residents’ psychosocial well-being by fostering a sense of belonging and significance [57].

The contextual factors are considered complementary to the initial programme theory. Some aspects, e.g., changes in resident characteristics, require changes in implementation that go beyond the adaptive tailoring of complex programmes and can undermine the functionality of the care concept. More severe cognitive and physical impairment, as well as challenging behaviours, are associated with a higher need for direct care time [58]; thus, to be able to maintain the same quality of care, the care concept needs systematic adaption to meet these changing needs [59]. Another contextual aspect that is prone to change is the organisational culture that depends on the leaders and is therefore vulnerable to personnel changes. As a result, person-centred care is not conceived comprehensively in the sense of a process of cultural development that takes into account the care environment, prerequisites and the macro context [52]; thus, its effect cannot reach its full potential [60].

Our results provide useful insights for various stakeholders in nursing homes in the process of planning, managing, controlling and monitoring practice development projects, especially but not exclusively in dementia care. As already mentioned by Lawton [17], the congruence between the elements of a care concept, the context and the needs and abilities of persons with dementia is crucial for achieving desired outcomes. In the care concept studied, the combination of the development of the skills and knowledge of nurses, the physical environment, the nursing time resources, and the leadership style is congruent. Our results also show that for the sustainable implementation of the care concept, shared values, and a long-term plan together with a systematic implementation process are beneficial.

The results of the process evaluation that confirm, complement or modify assumptions of the initial programme theory are used together with those of the outcome evaluation to create a revised programme theory [26]. This study shows that complex programmes rely on mechanisms that can be unexpected and that elude initial assessment by researchers. Therefore, it is advisable to include empirical data in the development of programme theories of complex programmes [61, 62]. To further develop the theoretical understanding of dementia care in long-term care, future research should pay more attention to the questions of what works for whom under what circumstances [63] and thereby contribute to an overarching understanding of the active ingredients and their mechanisms of impact of specialised dementia care.

### Methodological discussion, strengths and limitations.

The current study provides an elaborate example of a process evaluation within the approach of theory-driven evaluation in nursing science that focuses on mechanisms of impact and contextual aspects. Even though an increasing number of evaluation studies are incorporating process evaluation, most assess programme fidelity [64–68]; few go beyond this aspect and illuminate the underlying mechanisms of complex programmes. To assess the mechanisms within process evaluation, they use different approaches to theory-driven evaluation, realist evaluation or no theory or methodology at all [69–71]. The care concept of the SCU was developed by practitioners and guided by their experience, and the initial programme theory was based on their assumptions and research evidence. However, the changes, extensions and refinements to the initial programme theory yielded by the process evaluation show how important empirical data from the specific context are for understanding the mechanisms [62]. We gathered data from different contexts, namely, from SCUs and NSNHs, and the constant comparison of the contexts and the interventions, along with a qualitative data-driven research paradigm, enhanced the understanding of the potentially unknown active ingredients of the care concept.

Complex programmes such as care concept of SCUs pose almost infinite uncertainties [72]. Knowing that an all-encompassing assessment is not possible, we followed a pragmatic approach and focused on key uncertainties in our data collection and analysis. Furthermore, the outcomes of an SCU, especially those of higher order, such as belonging, can be attributed to several preconditions, only some of which are influenced by the intervention. Accordingly, a change in outcome can be caused by the intervention or a contextual aspect. This is because data on outcome indicators should be collected at all stages of the outcome chain of the theory of change [27]. Complex programmes are influenced by infinite contextual aspects. As we could not examine all of the contextual aspects, our study focused on those aspects that showed a substantial influence on the care concept in the inductive research process [32].

Further strengths and limitations of the study are based on the complexity of the care concept, the resulting methodological requirements and the implementation of the concept in only two SCUs. The qualitative inductive approach to process evaluation, which includes different perspectives and data collection methods, is considered a strength that is reflected in the mechanisms that only become apparent in the in-depth exploration of the care concept. Thus, the validity of the results is limited to the context of this care concept. As other SCUs involve other interventions in other contexts, the current results are not transferable but rather provide information about potentially relevant mechanisms, outcomes, and contextual aspects. However, the application of a theory-driven approach to guide the design of an evaluation [26] strengthens the accountability, construct and internal validity of the evaluation [73].

## Conclusion

The current study provides an example of a process evaluation that focuses on mechanisms, outcomes and contextual aspects and thereby assesses how an intervention brings about change. It contributes to understanding what the active ingredients of SCUs are and how they work. The care concept of the SCUs contributes to increased competence and self-efficacy of nurses, and higher levels of relaxation, purposeful activities and social interaction of residents. The implementation of such a concept highly depends on the skills of the nursing team and their shared understanding of nursing practice. As the organisational culture is shaped by individuals and is lacking systematic development, it is comprehended as fragile and vulnerable to unintended changes. Additionally, changes in resident characteristics affect the implementation of the care concept and its outcomes. With further research from different contexts, the findings can be consolidated, and thus, a higher-level understanding of the effects of specialised dementia care can be gained. These findings are important to the further development of dementia care practices on a sound theoretical basis.

## Data Availability

The datasets used and analysed during the current study are available from the corresponding author on reasonable request.

## Abbreviations

NSNH: Non-specialised nursing home
SCU: Special care unit

## Footnotes

1 Validation Training: Validation Therapy is trained in three-day basic courses and certification courses Level 1 to 4. Level 1 (Validation Worker) lasts 9 months and includes five, 2-day theory sessions, practice phases and supervision sessions. Level 2 courses (Validation Group Leader) last six to 9 months and consist of three, 2-day theory sessions, practice phases and supervision days [22].

2 Elder-care (Seniorenbetreuung): The elder-care team consists of individuals with a formal training in the promotion of activities, occupation and companionship for the elderly. In these nursing homes, they provide group activities for the residents on a daily basis scheduled in a weekly plan. They are not part of the nursing staff.
